# The Use of Induction Sensors and Helmet-Based Shielding Technology to Identify Differences in Electromagnetic Fields in Patients With Cranial Neurological Disease Versus Healthy Controls

**DOI:** 10.7759/cureus.45361

**Published:** 2023-09-16

**Authors:** James Brazdzionis, Maxwell A Marino, Imran Siddiqi, Dan E Miulli

**Affiliations:** 1 Neurosurgery, Riverside University Health System Medical Center, Moreno Valley, USA

**Keywords:** neural circuits, traumatic brain injury, brain mass, hemorrhagic stroke, stroke, emf, sensors, electromagnetic field

## Abstract

Background and objective

Electromagnetic fields (EMFs) stemming from neural circuits have been evaluated in healthy human subjects by using non-invasive induction sensor technologies with adjunctive shielding constrained to a helmet constructed of Mu-metal and copper mesh. These EMF measurements have been analyzed and discerned to alter physiological states of movement, thoughts of movement, emotional thoughts, and planned activities. However, these technologies have not yet been investigated as a diagnostic tool in patients with cranial neurological pathology to evaluate differences in patterns in the pathologic state compared to healthy controls. In light of this, we conducted this study to address this scarcity of data.

Methods

An observational study was conducted in which patients at a single center with cranial neurological disease of all causes were eligible to enroll; they had real-time non-invasive continuous EMF measurements obtained using induction sensors and a shielded helmet. These measurements were obtained in the resting state and then compared to previously obtained measurements in healthy volunteers. Post-processing analysis was conducted to evaluate the derivatives of these EMFs to identify changes in patterns.

Results

Fourteen patients with traumatic injury, stroke, and neoplasm with ages ranging from 14 to 81 years underwent successful analysis and post-processing of their cortically generated EMF waves. Patterns of EMF waves were compared to previously obtained data from four healthy controls. It was identified that there was less variation in the EMF measurements in patients with neurological disease compared to healthy controls. This was identified based on differences in derivatives of the EMF waves and decreased numbers of peaks and valleys in the EMF waves.

Conclusions

Novel induction sensors with an engineered, layered Mu-metal and copper mesh helmet for shielding with Mu-metal EMF channels appear to be efficient in measuring neural circuit-driven EMF non-invasively, in real-time, and continuously and can discern differences in EMF patterns between healthy volunteers and patients with neural circuit pathology. The decreased variability in EMF measurements in patients with neural pathology and greater decreases in slope within low-frequency measurements may be correlated with disrupted neural signaling from dysfunctional neurons and abnormalities in spatial and temporal summation. Some EMF changes in ill individuals correspond to changes in the experimentally induced lesions in the animal model. Further studies are warranted to devise models of disease and healthy states to improve these technologies as a diagnostic modality.

## Introduction

Neural electromagnetic fields (EMFs) have been appropriately evaluated in human subjects in real-time, in a non-invasive and continuous manner by using a novel lightweight portable helmet [1-5]. Similarly, it has been found that induction sensors within this configuration have the sensitivity to discern differential activities such as emotional thoughts, planned activities, and motor behavior from baseline resting states at a distance [1-5]. Previous shielding technology such as magnetoencephalography (MEG) has limited applications in terms of EMF measurements due to reliance on large-scale shielded rooms requiring research on reducing costs and space requirements associated with shielding [3,6,7].

Similarly, in human subjects, this lightweight portable helmet sensor technology has only been utilized in healthy individuals without known cortical pathology [1-5]. However, from a clinical perspective, if this technology can be used to evaluate abnormal neural circuits at the cortical and subcortical levels, there would be potential utility as a diagnostic modality. It is known that EMF abnormalities occur throughout different disease states through abnormalities in MEG [8-11]. These abnormalities have not been identified using this novel sensor and helmet-based technology. This helmet-based technology is ultra-portable with shielding constrained to a dual-layered Mu-metal (MuMETAL, Magnetic Shield Corporation, Bensenville, IL), interlaced copper mesh, and an air gap.

The helmet is engineered with ports with spacing to place EMF channels constructed with Mu-metal. Sensors are placed within the EMF channel to create shielding around the sensor and isolate the signal of the subject within the lightweight helmet. Unused ports have plugs placed within them constructed with layers of the Mu-metal, copper mesh, and containing an air gap to allow for continuous shielding of external EMF. This helmet is designed such that it can be worn continuously within a variety of settings and has been found to exclude “noise” EMF signals from the external environment without the need for a shielded room [3]. As such, clinically, this has the potential to be applied in a point-of-care setting in the clinic, at the bedside within the ICU, ambulance, or emergency department. Associated induction sensors (BS-1000, Quasar Federal Systems, San Diego, CA) are constructed to measure EMF in real-time, continuously, and non-invasively, and have been used in previous studies [1-5]. Therefore, it was hypothesized that by using these induction sensors and a helmet it might be possible to evaluate pathologic EMF measurements in brain-injury patients with any neurological disorder and distinguish abnormal pathology from normal pathology. Patterns of abnormality would need to be investigated to identify whether this modality is efficient.

## Materials and methods

The design of this study was approved by the Arrowhead Regional Medical Center Institutional Review Board with protocol number 21-05. This project was a cross-sectional observational trial in which patients with cranial-based injury or pathology who were admitted to the hospital and cared for by the neurosurgical service were eligible to participate and had measurements obtained of their neuronal EMF. The inclusion criteria were as follows: all neurosurgical patients with a cranial pathology; and patient recordings would take place at the bedside within an ICU setting. These patients would be compared to four healthy subjects previously evaluated during preliminary research by using the helmet and sensors and reported elsewhere [2,3,5]. These four healthy subjects were all over the age of 18 years (three male subjects and one female subject). The patients who declined to participate were excluded from the study.

After obtaining consent, 16 patients were initially assessed with EMF measurements recorded. Of them, 15 had complete readings measured with one having technical errors in measurement that were not identified until post-processing, and one patient with complete recordings had an incomplete record. These two patients were excluded from the analysis. Diagnosis, clinical status as measured by the Glasgow Coma Scale (GCS) score, and etiology of injury were assessed and classified into subcategories: traumatic injuries, malignancy, and stroke. Traumatic brain injuries included any injury due to traumatic events. These injuries included gunshot wounds to the head, skull fractures, subdural hematoma, epidural hematoma, traumatic subarachnoid hemorrhages, and contusions, all confirmed with imaging. Strokes were categorized as injuries related to spontaneous intracranial hemorrhage including cortical and subcortical hemorrhage, and spontaneous intraventricular hemorrhage. There were no cases of spontaneous or aneurysmal subarachnoid hemorrhage. One patient was diagnosed with a tumor in the form of a meningioma by imaging and subsequent surgical pathology. Patients were included in the study irrespective of whether they had undergone surgical intervention or not. Demographic data including age, sex, and race were recorded based on data imported into the electronic medical record.

An engineered dual-layer Mu-metal shielded helmet with interlaced copper mesh was acquired as in previous studies [1-5]. The helmet used in this study was a remanufactured model with an outer and inner layer of plastic surrounding the Mu-metal, air gap, and copper mesh layers. Within the helmet, ports were created during manufacturing for the placement of EMF channels and induction sensors [2,3]. Unused ports had a plug placed, which was constructed with the same functional layers utilized in the helmet with Mu-metal, copper mesh, and an air gap to create continuous EMF attenuation. Induction sensors were placed within the helmet oriented with sensor Bx in the left temporal region, sensor By in the right temporal region, sensor Bz in the right frontal region near the right primary motor cortex, and sensor B319 in the left frontal region near the left primary motor cortex, all with positive ends pointed towards the scalp. The helmet was suspended from an apparatus to support the helmet in position over the subject’s head, thereby preserving sensor orientation while reducing patient movement and helmet slippage and ensuring consistent orientation from subject to subject.

After data recordings were passed through a low pass filter (2kHz) with a 10x gain module, EMF measurements were acquired using a 16-bit National Instruments data card (National Instruments Corporation, Austin, TX) to obtain signals from 1Hz to 2kHz with a manufactured detection sensitivity of 1pT/√Hz. Data were binned into 20-second evaluated segments, allowing for the recording of 100,000 data points/Hz. Binned data were analyzed using a fast Fourier transform (FFT) algorithm with Igor® Pro version 8 (WaveMetrics, Lake Oswego, OR) for specific evaluation of the frequency domain. EMF patterns were quantitatively and qualitatively assessed for morphologic patterns in waveforms, similar peaks (defined as the maximal amplitude at a frequency greater than the adjacent amplitudes for adjacent frequencies), and valleys (defined as the minimum amplitude at a specific frequency where the adjacent amplitudes are greater than the evaluated data point). Within the waveform analysis, careful attention was paid to slopes to evaluate for changes in EMF patterns using the Igor® Pro software, and derivative equations were utilized to better plot the characteristics associated with each waveform. The recordings diminish from higher voltage to lower due to the Y-axis, the amplitude being the voltage divided by the square root of the frequency. Thus, the higher the frequency, moving along the X-axis, the lower the amplitude. The data were compared through analysis of waveforms to previously published data in the lesioned animal model obtained with the same portable lightweight helmet [12-14].

## Results

A total of 14 patients were assessed. The mean age of the assessed patients was 54.29 years old, and they had an average GCS score of 10.86. There were 10 male and four female patients. Clinical characteristics including the etiology of injury classified into traumatic injury, malignancy, and stroke are presented in Table 1.

**Table 1:** Characteristics of study participants

Age, years	Sex	Race	Pathology side	Etiology	Glasgow Coma Scale score
59	Female	Caucasian	Left	Stroke	13
66	Female	Caucasian	Right	Stroke	15
61	Female	Hispanic	Midline	Stroke	15
70	Male	Hispanic	Right	Stroke	5
72	Female	Caucasian	Left	Stroke	5
66	Male	African American	Right	Stroke	13
55	Male	Hispanic	Left	Traumatic	15
14	Male	Hispanic	Right	Traumatic	14
47	Male	Caucasian	Left	Traumatic	4
81	Male	Caucasian	Left	Traumatic	15
50	Male	Hispanic	Left	Traumatic	9
41	Male	Hispanic	Right	Traumatic	11
20	Male	African American	Bilateral	Traumatic	3
58	Male	Hispanic	Left	Malignancy	15

Electromagnetic field measurements

**Figure 1:**
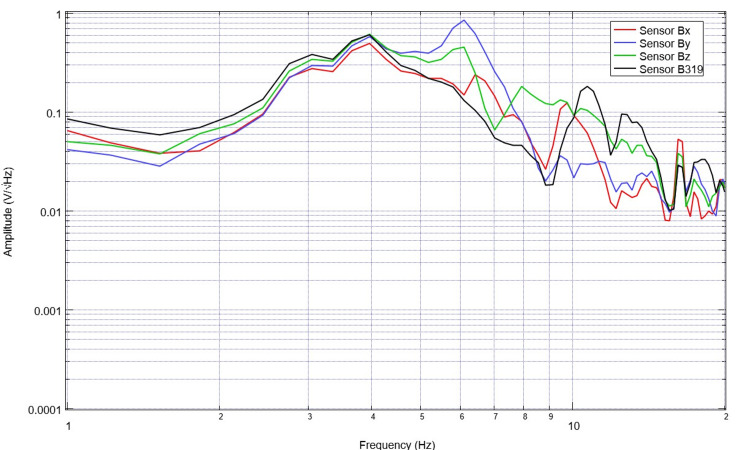
Normal electromagnetic field measurements of a healthy subject Normal electromagnetic field measurements of a healthy subject were assessed. We observed prominent and overall relatively evenly distributed peaks and valleys with variation in slopes of the electromagnetic field waveform

**Figure 2:**
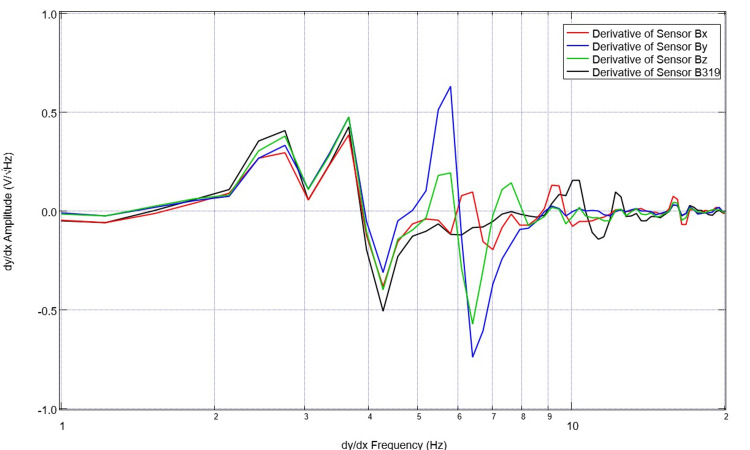
Normal first derivative of electromagnetic field measurements of a healthy volunteer The first derivative of the health subject measured in Figure 1 was plotted. We observed large-scale changes in the derivative throughout the investigated frequencies

Healthy volunteers were previously assessed in alternative investigations and trials completed by the investigators. Their data were utilized for assessment as control data [1-5]. An example of the normal control EMF is seen below in Figure 1. Similarly, the derivative of this subject’s EMF waveform was obtained using post-processing to better understand the rates of change of EMF measured. This is plotted in Figure 2.

**Figure 3:**
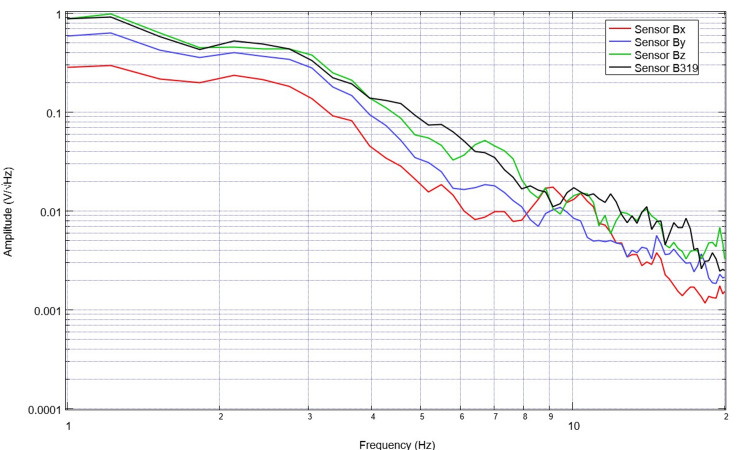
Electromagnetic field measurements from neuronal activity in a patient with a left-sided basal ganglia hemorrhage with intraventricular extension Electromagnetic field measurements were plotted for a patient with a left basal ganglia hemorrhage. The patient was comatose with a GCS score of 5. We noted fewer peaks and valleys in this patient’s waveforms with much less abrupt changes in slope when compared to the healthy subject GCS: Glasgow Coma Scale

**Figure 4:**
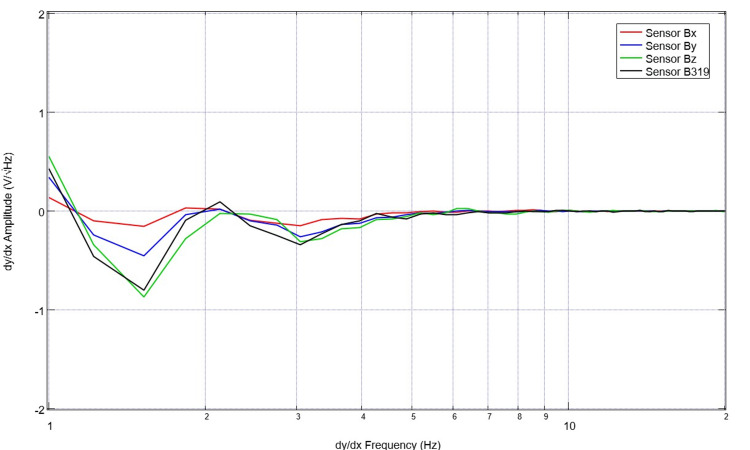
Plotted derivative of the measured electromagnetic field generated by cortical circuits in a patient with a left-sided basal ganglia hemorrhage with intraventricular extension Measurements were obtained from the evaluation of the derivative of the measured electromagnetic field generated by cortical circuits in a patient with a left-sided basal ganglia hemorrhage with intraventricular extension. The patient was comatose with a GCS of 5. The derivative identifies an early change in slope prior to two hertz, but as the graph increases along the X-axis, the changes are much less significant GCS: Glasgow Coma Scale

Similarly, the subjects’ neural circuits were assessed to evaluate those specific EMFs. An exemplary version of these signals is seen in Figure 3 from a patient with a left-sided basal ganglia hemorrhage with intraventricular extension. This patient was comatose with a GCS score of 5. The derivatives of these waveforms were also obtained using post-processing techniques to evaluate rates of change in EMF. This is plotted from the same patient, as shown in Figure 4.

**Figure 5:**
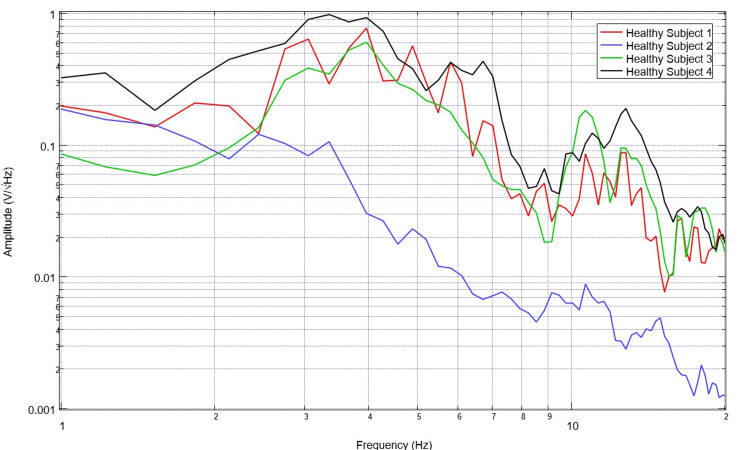
Comparison of neural electromagnetic fields measured through sensor B319 for all healthy volunteers Healthy volunteers are overall noted to represent a pattern with distinct peaks and valleys with abrupt changes in slope as well as relatively evenly spaced peaks and valleys in the measured electromagnetic field

**Figure 6:**
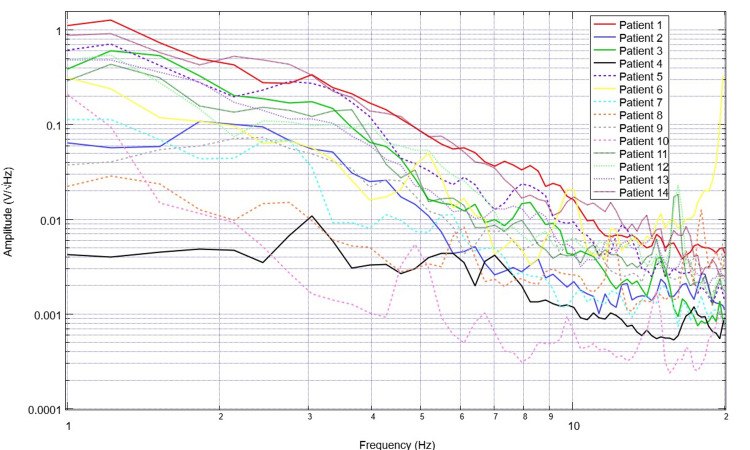
Comparison of neural electromagnetic fields measured through sensor B319 for all ill subjects Patients overall are noted to have much less defined peaks and valleys than healthy subjects with less variation in peaks and valleys due to a lack of large-scale changes between the peaks and valleys within the measured electromagnetic field generated by the cortical circuits

**Figure 7:**
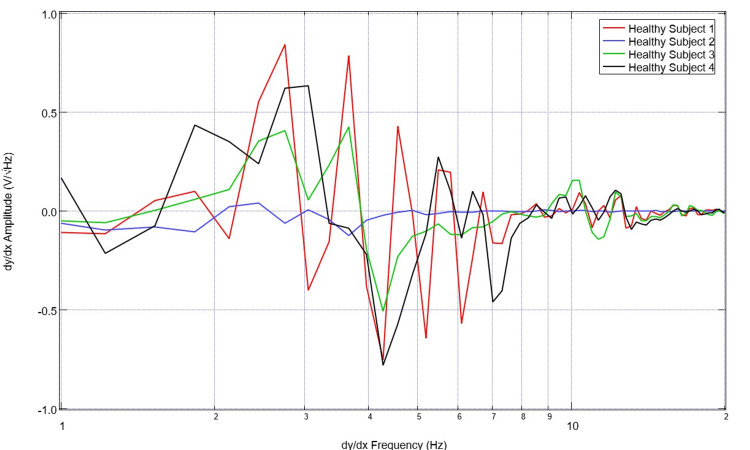
Comparison of calculated first derivatives from neural electromagnetic fields measured through sensor B319 for all healthy subjects Overall patterns identify consistently large-scale changes in the derivative with frequent changes from positive to negative and sharp, defined waveform morphology

**Figure 8:**
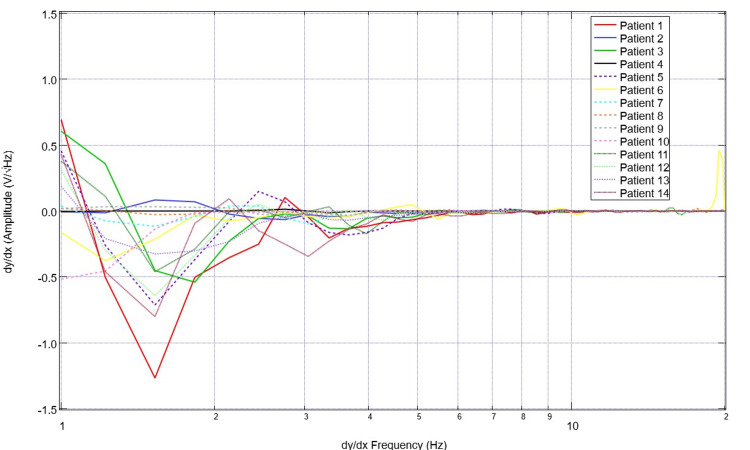
Comparison of calculated first derivatives from cortical electromagnetic fields measured through sensor B319 for all ill patients Derivatives of the electromagnetic field measured in patients overall had much less defined morphology than healthy volunteers with a much flatter waveform as the X-axis increased and much more negative slopes in frequencies less than 2 Hz

**Figure 9:**
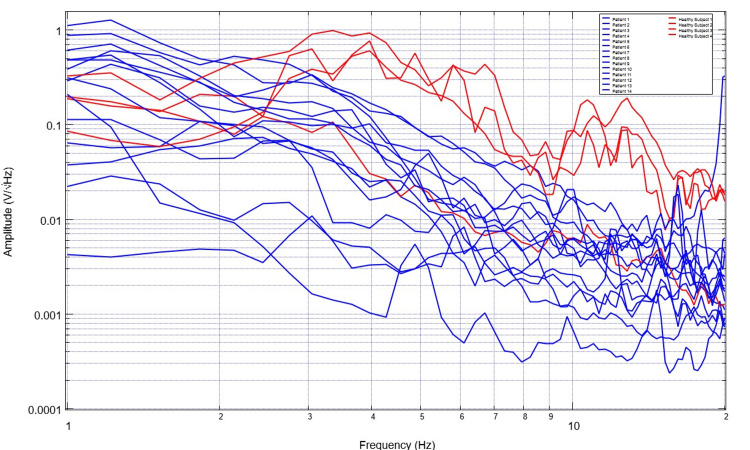
Comparison of neural circuit-related electromagnetic fields measured through sensor B319 for all healthy subjects versus ill patients A comparison of neural circuit-related electromagnetic fields measured through sensor B319 for all healthy subjects (red) versus diseased patients (blue) was plotted. Patients are demonstrated to have much less defined waveforms overall with less changes from peaks to valleys and more consistent slopes without abrupt changes in amplitude

**Figure 10:**
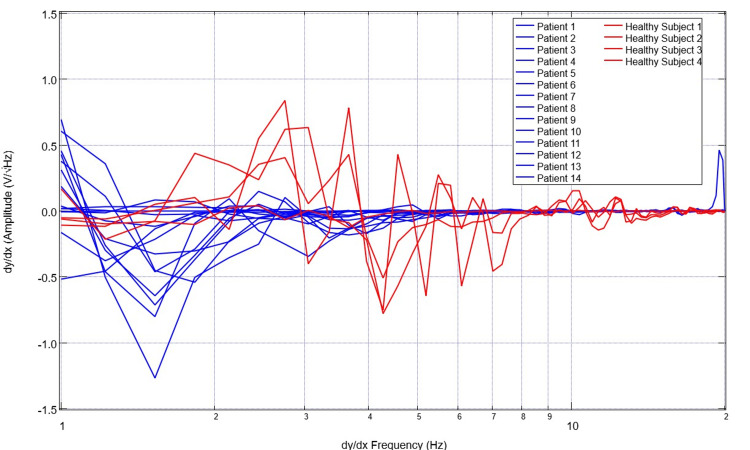
Comparison of calculated first derivatives from neural electromagnetic fields measured through sensor B319 for all healthy subjects versus ill patients The first derivative functions of measured cortical electromagnetic fields for healthy subjects (red) were plotted against patients (blue). It was noted that the patient derivatives all hover around the 0 mark as the X-value increases while healthy subjects have much more defined waveforms. This identifies regular and large-scale fluctuations in slope in healthy subjects compared to less fluctuations with overall less peaks and valleys in patients with neurologic injury

To assess for differences in neural circuitry, EMF measurements from singular sensors were assessed. To compare data optimally, a single sensor was evaluated to create a more readable plot and to control for the different sensor orientations and subsequent EMF signals. Sensor B319 was selected for analysis to consistently evaluate a singular cortical region. Evaluation of EMF measurements from the selected healthy subjects is plotted in Figure 5 and that of patients with disease is plotted in Figure 6. The derivatives of these measurements are evaluated in Figures 7-8. Similarly, the EMF measurements for all patients were plotted against the healthy subjects (Figure 9), as were the derivatives of these two groups (Figure 10).

Several patterns were noted in these data sets. Patients with neurologic injury had variable presentations and levels of consciousness ranging from GCS scores of 3 to 15 and bilateral presentations of injury. Therefore, the morphologic changes of injury were highly variable within their associated EMF measurements. However, it was noted that patients with neurologic pathology had rapidly decreasing slopes early on their graphs within the low frequencies from 1-3 Hz compared to the healthy subjects. It was also noted that the EMF measurements in patients with neurological pathologies had fewer variations in their associated waveforms with overall fewer peaks and valleys, wherein peaks are defined as a maximal point on the Y-axis where adjacent Y-values for adjacent points are lower than the investigated point. Valleys are the minimum Y-values with adjacent Y-values that are higher than the incident point.

Similarly, evaluating the derivatives yields an interesting observation. As seen in Figure 10, it appears that patients with neurologic pathology largely have greater negative derivatives in early derivatives of frequency regardless of whether or not they had undergone surgical intervention as many patients in this population group had not undergone surgical intervention and many were treated nonoperatively, especially those that were in the stroke and traumatic population with GCS scores greater than 8. Additionally, we noted large obvious peaks and valleys with high amplitude changes of the derivative graphs in healthy patients through 7Hz. This identifies high degrees of variability in slope and therefore EMF measurements within the healthy subjects compared to a less variable pathologic state. These evaluations were compared to previously published cortical impact animal models for EMF changes [12-14].

## Discussion

This study was conducted to evaluate the overall efficacy of EMF measurements using a novel EMF-shielded lightweight portable helmet constructed with Mu-metal and copper mesh and EMF channels (Mu-metal) with novel induction sensors to discern neuronal circuit pathology in ill patients compared to healthy subjects. Patients with variable neurologic pathology ranging from tumor, brain trauma, and stroke were evaluated. It was noted that one patient in this study had a tumor, while others had brain trauma ranging from small traumatic subarachnoid hemorrhages with little to no neurologic deficit (GCS=15) to a large gunshot to the head in a patient with a GCS of 3.

Evaluation of healthy subjects has previously been conducted with these technologies. However, to our knowledge, these specific techniques have not yet been applied to clinical pathologic states in humans [1-5]. In this study, an evaluation of the morphologic characteristics of the EMF waveforms and their associated derivatives was conducted to identify patterns in a similar fashion to analysis conducted with electroencephalography where patterns are utilized for diagnosis and evaluation of specific dysfunction. Specifically, waveform derivatives were evaluated to describe the slope of the evaluated waveform to identify its degree of variance.

Effectively, decreases in variability identify a difference in signaling pathways and alterations in neuronal functioning. Physiologically, electrical currents drive the development of a magnetic field due to Farraday’s law. Similarly, changes in an electrical current should cause changes in magnetic fields and field measurements. As it is known that neural excitability is related to electrical currents causing depolarization or hyperpolarization, cellular injury should change the appropriateness of these depolarizations or repolarizations [15]. Correspondingly, in the normal state when neurons from altering locations fire, magnetic fields should be manipulated and changed dynamically. These dynamic changes result in variability within EMF. Therefore, with injury, it is thought that with decreased efficacy in electrical signaling, there must be decreases in magnetic field changes. These changes alter the abilities of cells to participate in temporal and spatial summation causing neurologic deficits. Therefore, these measured decreases in variation may represent neurological injury itself as this was seen in nearly all the ill patients regardless of their GCS score. Similarly, this corresponds to the existing theory of consciousness wherein variations in neural activity through the temporal variation of electrical charge and therefore EMF may represent consciousness and neurologic function [16]. Further studies are required about the use of artificial intelligence (AI) models to better understand these variations in physiologically normal and pathologic states to predict injury.

It is known that trauma, whether from stroke, physical injury, or neoplasm, causes direct cellular injury albeit with differing mechanisms. These direct cellular injuries cause abnormalities in function within the cells, which necessarily cause these abnormalities in EMF measurements. Although these abnormalities in EMF measurements were measured in-vivo with imaging correlation, pathologic cells were not obtained in this human observational study. There may be a need for further human investigations correlating sensor EMF changes to histology on a cellular and subcellular level. Further studies may endeavor to histologically and through electrophysiological means evaluate human cells and their functionality as well as correlate the EMF measurements obtained from injured cells to develop a better understanding of the functional effects of these disease processes. These investigations have been initiated in alternative models. In fact, the original investigation of the ability of this sensor to identify an EMF was in neonatal mice hippocampal cells with histological comparison [17]. Subsequent studies have identified an animal cortical impact model that demonstrated specific EMF changes corresponding to histological changes and biomarker changes for brain trauma [12].

In the animal cortical impact model studies, EMF changes were seen, but only after injury, with analogous changes to the pathological EMF changes in this human observational study [12-14]. These changes involved a downslope occurring between 1.4 Hz and 2 Hz, an overall flattening of the waveform with fewer peaks and valleys from 4-6Hz, and changes in patterns of peaks and valleys between 10-12 Hz, which correlate to the changes seen at similar frequencies in the animal models with changes at 2.5 Hz, 6.5 Hz, 11 Hz decreased variation of EMF measurements post-TBI between 4.5 Hz and 7 Hz [12-14]. In the cortical impact animal model, those changes corresponded to the histological changes and a change in brain trauma biomarkers [12]. Furthermore, in the cortical impact animal model, when the damaged individual animals had specific targeted EMF frequencies applied through stimulation, either delayed after two days or after 20 minutes, there was an identified return towards normal pre-injury EMF recordings [12,14]. The return to normal was greater when the specific stimulus was applied 20 minutes after injury and was seen both physically, behaviorally, histologically, and in measured biomarkers [12].

Since in the animal model identifying specific EMF frequency changes after injury allowed specific application of an EMF stimulus, the changes in this observational human study may be extrapolated to identify and then apply a specific EMF stimulus to improve function, behavior, and possibly improve histology and biochemically. Similarly, due to the efficacy demonstrated through these animal models, it is thought that these principles of stimulation may be considered for future applications in research for the treatment of neurological disease states. There are several studies that utilize similar technologies that are not as specifically targeted with appropriate safety, tolerability, and potential efficacy for these disease states [18-21]. It is hypothesized that targeted stimulation with EMF frequencies and stimulation targets directed at the area of interest using applied data will demonstrate similar safety profiles with the potential for improved treatment efficacy in future trials. Therefore, it is thought that the data presented from this project overlapping with animal studies, and with identified differences between healthy volunteers and patients with neurological pathology, may aid in the development of future large-scale studies investigating these novel technologies in the form of the development of diagnostic models and development of guided treatment protocols to evaluate the efficacy of targeted EMF modulation on neural pathology.

It is important to note that the measurements obtained in this study were acquired in real-time, with continuous and dynamic measurements of the EMF evaluated non-invasively in a non-contact fashion. Since these sensors demonstrated efficacy in identifying a difference in pathological states from normal healthy controls, and activity and emotion within normal states, this identifies a critical use where this technology may continue to be developed as a point-of-care diagnostic tool. This system is portable due to its constrained system of a helmet with sensors connected to a laptop. This would allow for direct point-of-care use in a variety of settings ranging from pre-hospital, emergency departments, and ICUs, to the clinic, and outpatient use. Furthermore, with improved AI modeling, it may be possible to develop predictive models of EMF that identify specific regions and degrees of dysfunction. This increased diagnostic utility with these models could have wide-ranging clinical implications enabling rapid triage to point-of-care diagnosis in unstable patients. Further research is required to develop these models.

Limitations

This study had a relatively small sample size, comprising only 14 patients and four healthy volunteers. Further studies need to be conducted with larger sample sizes to evaluate the reproducibility of our findings. Additionally, there are limitations in evaluations of specific disease states due to low sample sizes as noted, with only one evaluated patient having a tumor. Furthermore, stratifications of preoperative and postoperative evaluations and usage of antiepileptic drugs may be investigated in future studies. Larger-scale studies are necessary to better correlate measurements clinically. Similarly, analysis of these waveforms was conducted by evaluating overall patterns in morphology in EMF measurements and derivatives as representations of the slope or changes in EMF. The development of models of these patterns using AI techniques may enable further applications and understanding. Similarly, analysis of cellular and subcellular processes may be considered, if possible, to correlate the physiologic measurements of EMF and imaging with the ongoing cellular processes in these patients.

## Conclusions

Novel induction sensors (model BS-1000, Quasar Federal Systems, San Diego, CA), an engineered, layered Mu-metal and copper mesh lightweight helmet for shielding, with Mu-metal EMF channels, can effectively measure neural circuit-driven EMF non-invasively, in real-time and continuously, in healthy volunteers and patients with neurologic pathology. Additionally, patterns of neural-driven EMF vary between patients with symptomatic and imaging-correlated pathologic processes ranging from stroke and trauma to tumor and normal controls. There is less variability in individual EMF measurements in patients with neural pathology compared to healthy controls and greater decreases in slope within low-frequency measurements. This phenomenon may represent disruption in cellular and subcellular signaling resulting in dysfunction in spatial and temporal summation. Further studies are warranted to produce models of disease and healthy states to improve these technologies as a diagnostic modality and for investigation of the potential of EMF modulation and stimulation as a treatment modality for neurological disease states.
